# Comparison of Labrum Resistance Force while Pull-Probing In Vivo and Cadaveric Hips

**DOI:** 10.3390/biomimetics6020035

**Published:** 2021-05-31

**Authors:** Takehito Hananouchi, Soshi Uchida, Yusuke Hashimoto, Funakoshi Noboru, Stephen K. Aoki

**Affiliations:** 1 Medical Engineering Laboratory, Department of Mechanical Engineering, Faculty of Engineering, Osaka Sangyo University, Daito, Osaka 574-8530, Japan; 2 Department of Orthopaedic Surgery, Shimada Hospital, 100-1 Kashiyama, Habikino, Osaka 583-0875, Japan; 3 Department of Orthopaedic Surgery, Wakamatsu Hospital of University of Occupational and Environmental Health, Kitakyushu 808-0024, Japan; soushi@med.uoeh-u.ac.jp; 4 Department of Orthopaedic Surgery, Graduate School of Medicine, Osaka City University, Abeno-ku, Osaka 545-8585, Japan; hussy@med.osaka-cu.ac.jp; 5 Department of Orthopaedic Surgery, Kyoto Shimogamo Hospital, Kyoto 606-0866, Japan; funaboo2001@yahoo.co.jp; 6 Department of Orthopaedics, University of Utah, Salt Lake City, UT 84108, USA; stephen.aoki@hsc.utah.edu

**Keywords:** hip labrum, tissue resistance, probing device, cadaver tissue

## Abstract

Cadaver tissue has been identified as the highest-fidelity anatomical representation in terms of the training for orthopedic surgery, including for arthroscopy of a damaged hip labrum. However, hip labrum stiffness in vivo and in cadavers has not been directly compared. The purpose of this study was to compare in vivo and cadaveric hip labrum stiffness during pull-probing with a force sensor. We measured the resistance force of the hip labrum in ten patients during hip arthroscopy (i.e., in vivo) and compared it with ten cadavers, both intact and detached from the acetabulum, using a surgical knife. We confirmed a partial labral tear (i.e., not detached fully from the rim) at an antero-superior potion in all of the patients. The mean highest resistance levels for the hip labrum in the patients (4.7 N) were significantly lower than the intact cadaveric labrum (8.3 N), and slightly higher than the detached labrum (4.2 N). In this study, the stiffness of the cadaveric labrum tissue was similar to that of the in-vivo hip labrum.

## 1. Introduction

Hip arthroscopy is an innovative surgery addressing intra-articular pathologies of the hip joint, especially femoroacetabular impingement (FAI), which was recently unveiled [1]. Hip arthroscopy is technically challenging, and surgical training is mandatory in order to improve surgical skills and minimize surgical time and potential complications [1]. 

In terms of surgical training for hip arthroscopy, fresh cadaver tissue has been identified as the highest-fidelity representation of surgical anatomy, which is performed in vivo and is almost impossible to replicate in synthetic models [2]. Cadaveric simulation offers the opportunity to practice an entire operation with a similar environment, equipment, and psychological fidelity, thereby enabling the rapid acquisition of procedural skills [3]. The cadaver specimen has also been useful in the development of medical devices and biomimetic materials to reproduce in vivo operations [4].

However, recent literature lacks a clear understanding of whether the stiffness of the hip labrum in the cadaveric tissues is different from that in patients. The stiffness of the in vivo hip labrum can be measured and compared with that of the cadaveric hip labrum using a probing device with a tri-axial force sensor (Probing Sensor, Takumi Precise Metal Work Manufacturing Ltd., Yao, Osaka, Japan) [5,6] developed by one of the authors to quantitatively measure the resistance of soft tissues in the joint. 

The purpose of this study was to compare the stiffness of in vivo and cadaveric hip labrums during probing with this device. For this purpose, we measured the resistance of the hip labrum in patients undergoing hip arthroscopy and compared it with the resistance of the cadaveric labrum in both the intact (1) and detached (2) conditions.

## 2. Materials and Methods

Ten patients, including five men and five women, undergoing hip arthroscopic treatment for symptomatic cam-type FAI lesions participated in this study. The mean age (and standard deviation (SD)) was 41.5 ± 3 years in the age range of 23–60 years. All of the patients gave informed consent, and the study was ethically approved by institutional review board (please see Institutional Review Board Statement and Informed Consent Statement ) in the hospitals. Three surgeons from the list of authors performed the surgeries in the supine position on a traction table under general anesthesia with a well-padded peroneal post. An anterolateral portal was established under fluoroscopy, then mid [7,8] or modified anterior [9] portals were established manually, and an inter-portal capsular cut was performed to improve their accessibility. Using a normal metallic probe, we confirmed the presence of a partial labral tear (i.e., imperfect detachment of acetabular rim from the bone) at the anterosuperior portion in all ten cases. Then, the measurements were taken using the probing device [6]. 

**Figure 1:**
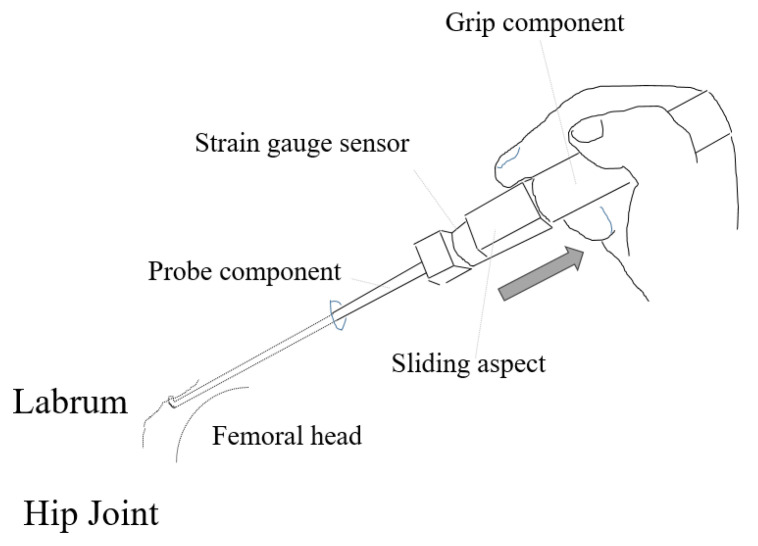
A scheme of the resistance force measurement of the hip labrum while pulling the device probe (Probing Sensor, Takumi Precise Metal Work Manufacturing Ltd., Yao, Osaka, Japan). The gray arrow indicates the pulling direction.

A sliding aspect with a strain gauge sensor in the probing device was used to manually control the distance along the probe axis with the surgeon’s index finger or thumb while pulling the probe component (Figure 1). The probing device could not record the sliding distance, but just determined it. the The sliding distance was set to 3 mm in this study [6]. Dedicated software visualized the three forces at the separate graphs in the x, y, and z directions (x was the transverse direction, y was the vertical direction (direction of the hook), and z was the probe axis) measured in real-time with a frequency of 50 Hz.

**Figure 2:**
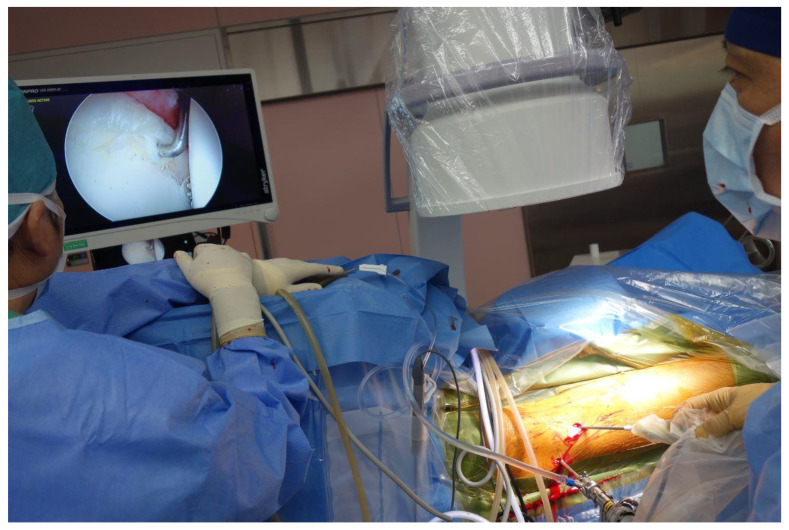
The surgeon (on the right) is measuring the resistance force of the labrum with the probing device. The process is directly observed on the monitor.

We placed an arthroscopic camera into the first anterolateral portal, then inserted the probing device through the anterior portal with a thin elastic cover normally used intraoperatively in an ultrasound device (Probe cover, CIVCO Medical Solutions, Coralville, IA USA) [5,6], and went further into the hip joint until the tip of the device was located below the inner side of the hip labrum. The measurements of the hip labrum resistance in the anterosuperior potion were performed three times per case through the second portal (Figure 2). These measurements were made by one of the authors; the data were recorded using the aforementioned software.

Ten fresh-frozen specimens of cadaver pelvis and proximal femur were used to simulate normal hip arthroscopy [5]. The donors included one man and nine women; the mean age ± SD was 60 ± 17 years (range: 35–88 years) at the time of death. All of the specimens were radiographically screened to confirm the absence of an osseous abnormality. One author performed all of the measurements. As the probing device used in the cadaver study was a prototype model of the above mentioned device, a pulling distance of the probing device was controlled 2.5 mm manually on the arthroscopy monitor (i.e., using a half distance of the 5-mm probe interval marking on the probe) [5]. The resistance forces of the intact (1) and detached (2) hip labrum of cadavers were recorded and were compared with the aforementioned patients’ labrums.

The maximum value of the force detected in each measurement was considered as the resistance level. For the statistical analysis, (1) the force in the direction of the probe axis (i.e., only z direction), (2) the resultant force consisting of the forces measured in the directions along and perpendicular to the probe axis (i.e., the resultant force of z and y directions), and (3) the tri-axial resultant force (i.e., the resultant force of z, y, and x directions) were compared using the Kruskal–Wallis test in order to check whether there were differences in the resistance force of the hip labrum in vivo and of that in cadaver hips in the above two situations.

## 3. Results

**Figure 3:**
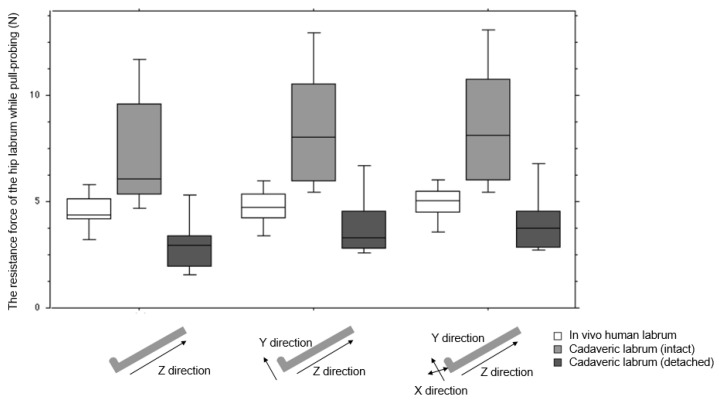
Resistance of the hip labrum in patients (white bar) and cadavers in the (1) intact (light gray bar) and (2) detached (dark gray bar) situations: along the probe axis (left, z direction), two-directional resultant resistance force along and perpendicular to the probe axis (middle, z, and y directions), and the three-directional resultant resistance force (right, all directions).

The differences in resistance forces between the patients and cadavers were statistically significant both in the axial direction (*p* = 0.0005) of the probe and in the two-axes (*p* = 0.0012) and three-axes (*p* = 0.0013) resultant force directions (Figure 3).

The mean (SD, 95% confidence interval (CI)) resistance force values in the direction along the probe axis (z direction) in vivo and in cadavers in situations (1) and (2) were 4.4 N (0.88, 3.9–5.0), 7.2 N (2.5, 5.7–8.8), and 3.0 N (1.4, 2.2–3.9), respectively. The mean two-directional (z and y) and three-directional (z, y, and x) resultant resistance forces were 4.6 N (0.89, 4.0–5.1), 8.2 N (2.7, 6.5–9.8), and 4.0 N (1.6, 3.0–5.0), and 4.7 N (0.89, 4.2–5.3), 8.3 N (2.8, 6.5–10.0), and 4.2 N (1.6, 3.2–5.2), respectively. 

## 4. Discussion

In this study, we investigated the resistance force of the hip labrum in vivo and in the intact and detached conditions of cadaver hips using a probing device, in order to clarify whether the cadaveric labrum can be used in surgical training, surgical simulation, and/or development of medical devices or biomimetic materials. 

There are several limitations of this study. First, the number of patients and cadavers we enrolled and used was small, their ages varied, and the gender was not perfectly matched. Secondly, we could not maintain the same conditions between the patients and cadavers. We could not test an intact labrum in the surgical patients given labral pathology noted at the time of surgery. In the cadavers, we measured two situations (an intact and a fully detached labrum). Thirdly, we did not use the same probing device in the in vivo operations and cadaver experiments. We could control the probing distance with the sliding part of the probing device in in vivo operations. On the other hand, we manually controlled the moving distance of the probing device on the arthroscopy monitor in cadaver experiments. Despite this difference, we consider that the results meet the purpose of this study. The discussion of the results points towards some tendencies regarding cadaver stiffness.

One of the most interesting findings in this study was that the resistance force of the in vivo hip labrum was in between those of the intact and detached situations of the cadaver tissue. For the representative result, the three-axial resistance force of the in vivo hip labrum was 4.7 N (95% CI; 4.2–5.3), less than the intact labrum (8.3 N (6.5–10.0)), and similar to the detached labrum (4.2 N (3.2–5.2)). The resistance force values followed the same order (detached, in vivo, and intact labrum) in two other cases. Following this result, one might assume that the cadaveric hip could be used for the surgical training of hip arthroscopy, including labrum repair.

Similar quantitative measurements of soft tissues while probing were made in two previous reports [10,11]. One of them [10] investigated the resistance force of meniscus tissue at pull-probing. Ten menisci were acquired from freshly frozen cadaveric knees and then mounted on a mechanical platform to monitor the force parameters. The maximum probing force was up to 8.5 N. In addition, the authors assumed that the cadaver tissue might be stiffer than living tissue, while the tissue from elderly people was not stiff as young and healthy people. Unfortunately, a direct comparison was not performed. Another paper [11] addressed a probing device equipped with a force and torque sensor. Several tests during knee arthroscopy, including pull-probing of the meniscus tissue, were performed in two surgical patients in the manuscript. However, there were no pull-probing force data reported, and a comparison between living and cadaver tissues was not made. To the best of our knowledge, a comparison between in vivo and cadaveric hip labrums has not been reported.

The quantitative value of the resistance force of the hip labrum determined using the probing device is a key parameter for the development of a surgical simulator or alternative biomaterial. Usually surgeons qualitatively judge on the basis of proprioceptive feedback using a metallic probe to check whether a target tissue is damaged. This study attempts to translate the surgical probing process into quantitative values. Therefore, the stiffness of a material or a biomimetic material can be quantified by the measured value of the resistance force of the target tissue. For example, one of the authors of this paper measured the resistance force of the hip labrum in a surgical training model [6]. The force value was 4.4 N (SD; 0.2 N) for the intact and 1.6 N (0.1 N) for the detached labrum. Therefore, the model tissue was softer than that in the in vivo human hip labrum. As the material of the labrum in the training model is made from rubber, a harder material should be used to mimic human labral tissue.

When the correlation between the resistance force value of the labrum while probing and the classical stiffness by a tensile tester device will be clarified, the resistance force value may be used instead of stiffness as a more meaningful and useful parameter. This is because the value of the resistance force can be converted to the one classical mechanical property, i.e., stiffness. Recently, it was clarified that the reaction force of articular cartilage surface while push-probing has a moderate correlation with the stiffness and elastic modulus determined by a micro-indentation device [12]. In a future study, the correlation between the resistance force value of the labrum while probing and the classical stiffness determined by a tensile tester device should be clarified.

## 5. Conclusions

Using a probing device to measure resistance force of soft tissues while probing, we measured the resistance force of the hip labrum in vivo in surgical hip arthroscopy patients, and in intact and detached labral cadaver hips, and showed that the stiffness of the cadaveric and in vivo tissues was similar. This information is important in order to facilitate surgical training, surgical simulation, and/or the development of medical devices or biomimetic materials. 

## Data Availability

Not applicable.
